# Predictors of poor outcomes after significant chest trauma in multiply injured patients: a retrospective analysis from the German Trauma Registry (Trauma Register DGU®)

**DOI:** 10.1186/s13049-014-0052-4

**Published:** 2014-09-03

**Authors:** Stephan Huber, Peter Biberthaler, Patrick Delhey, Heiko Trentzsch, Hauke Winter, Martijn van Griensven, Rolf Lefering, Stefan Huber-Wagner

**Affiliations:** 1 Department of Trauma Surgery, Klinikum rechts der Isar, Technical University Munich - TUM, Ismaninger Str. 22, Munich, D-81675, Germany; 2 IFOM – Institute for Research in Operative Medicine, University Witten/Herdecke, Faculty of Health, Ostmerheimer Str. 200, Cologne, D-51109, Germany; 3 Department of General, Vascular, Transplantation and Thoracic Surgery- Grosshadern Campus, Munich University Hospital (LMU), Marchioninistr. 15, Munich, D-81377, Germany; 4 Institute for Emergency Medicine and Medical Management, University of Munich, Schillerstr. 53, Munich, D-80336, Germany; Committee on Emergency Medicine, Intensive Care and Trauma Management (Sektion NIS) of the German Trauma Society (Deutsche Gesellschaft für Unfallchirurgie ,DGU), Munich, Germany

**Keywords:** Polytrauma, Blunt chest trauma, Severly injured, Outcome, Chest wall injury, ISS, Mortality, Ventilation

## Abstract

**Background:**

Blunt thoracic trauma is one of the critical injury mechanisms in multiply injured trauma victims. Although these patients present a plethora of potential structural damages to vital organs, it remains debated which injuries actually influence outcome and thereby should be addressed initially. Hence, the aim of this study was to identify the influence of critical structural damages on mortality.

**Methods:**

All patients in the database of the TraumaRegister DGU® (TR-DGU) from 2002–2011 with AIS Chest ≥ 2, blunt trauma, age of 16 or older and an ISS ≥ 16 were analyzed.

Outcome parameters were in-hospital mortality as well as ventilation time in patients surviving the initial 14 days after trauma.

**Results:**

22613 Patients were included (mean ISS 30.5 ± 12.6; 74.7% male; Mean Age 46.1 ± 197 years; mortality 17.5%; mean duration of ventilation 7.3 ± 11.5; mean ICU stay 11.7 ± 14.1 days).

Only a limited number of specific injuries had a significant impact on survival. Major thoracic vessel injuries (AIS ≥5), bilateral lung contusion, bilateral flail chest, structural heart injury (AIS ≥3) significantly influence mortality in study patients. Several extrathoracic factors (age, blood transfusion, systolic blood pressure and extrathoracic severe injuries) were also predictive of increased mortality.

Most injuries of the thoracic wall had no or only a moderate effect on the duration of ventilation. Injuries to the lung (laceration, contusion or pneumothoraces) had a moderate prolonging effect. Cardiac injuries and severe injuries to the thoracic vessels induced a substantially prolonged ventilation interval.

**Conclusions:**

We demonstrate quantitatively the influence of specific structural damages of the chest on critical outcome parameters. While most injuries of the chest wall have no or only limited impact in the study collective, injuries to the lung overall show adverse outcome. Injuries to the heart or thoracic vessels have a devastating prognosis following blunt chest trauma.

## Background

Blunt trauma to the chest and structural damages of vital organs inside the thoracic region have a substantial influence on morbidity and mortality for patients suffering from multiple injuries (polytrauma) Previous studies report about a mortality rate of up to 25% following severe thoracic trauma in polytrauma victims. In this respect, a recent report from the British Trauma Registry TARN has described a mortality of nearly 20% in 1164 patients sustaining blunt thoracic injury [1]. Recent publications obtained from data of the the German and British trauma registries described that blunt chest injuries are a serious medical and economic challenge in western industrial nations and that severe thoracic trauma can induce high mortality and prolonged ventilation periods.

However, due to the broad variance of influence of thoracic structures on prognosis, blunt thoracic trauma is a very heterogeneous entity. A number of scoring systems have been developed to evaluate the prognosis of patients following blunt thoracic trauma such as the Thoracic Trauma Score (TTS), Pulmonary Contusion Score (PCS) or the Wagner Score, which were calculated as independent indicators of prognosis considering mortality and morbidity following blunt thoracic trauma [2]. Moreover, age of 65 years or above has been described as a major predictor of mortality and required ventilation [3],[4].

Multiple injuries to the chest and its organs are found in the majority of patients after blunt thoracic trauma [1],[5].

However, for chest wall injuries such as rib fractures or flail chest, existing data remain controversial as to their prognostic impact, which is not consistent in prior investigations [3],[4],[6]. Some injuries such as major cardiac injury or injury of thoracic vessels are clearly immediately life-threatening injuries [7]. On the other hand, the impact of lung parenchymal injuries or pulmonary contusions also remains controversial in previous studies [8],[9].

Since the widespread introduction of CT scans to initial diagnostics in polytrauma patients, the sensitivity has increased and more injuries are identified immediately upon admission of patients after thoracic trauma [10]. Many structural damages are visualized in multi-slice computerized tomography (MSCT) as compared to conventional radiography in this collective [11].

Although it has been demonstrated that structural damages of intrathoracic organs may reduce the prognosis so far no clear quantitative ranking of the influence of specific injuries on morbidity and mortality were calculated. Hence, the aim of this study was to calculate the influence of specific anatomic structural damages in patients suffering from polytrauma with concomittant thoracic trauma on relevant outcome parameters. This study was performed on a large data collective of multiply injured patients, the TraumaRegister DGU® (TR-DGU).

## Methods

### Study design

This study was designed as a retrospective cohort study on data of trauma victims recorded in the national trauma registry of Germany (TraumaRegister DGU®). The observation period was from 2002 until 2011. Further details on the TraumaRegister can be found under the paragraph data collection.

To clarify the methodology of the study, we have divided methodology and results into three parts:

Part I results consist of patients demographic data as well as the influence of various demographic details on survival.

Part II results consist of a logistic regression analysis and forward selection of significant dependent variables to show the influence of various injuries on patient survival.

Part III results show the influence of various injuries on ventilator days in patients surviving the first 14 days after thoracic trauma as analyzed by multiple linear regression and stepwise forward selection. The deviation from the mean duration of ventilation in the sudy population is indicated as prolonged ventilation.

### Inclusion criteria in the study

Inclusion criteria were blunt trauma, ISS ≥ 16, sufficient data information to calculate the Revised Injury Severity Classification (RISC) score, AIS Chest of ≥2 and an age of 16 years or older at the day of injury.

Diagnoses following trauma are entered in a web based interface and stored in the form of the revised AIS score (2005 version). Diagnoses were identified in the data set by searching for the respective AIS code.

There was no specific funding for this study, It was completely financed by the Technical University Munich.

### Data collection

The data source for our study was the the TraumaRegister DGU®, which was started in 1993. It comprises data of major trauma patients of 266 trauma centers mostly from German-speaking countries (Germany, Austria, and Switzerland, but also The Netherlands, Belgium, and Slovenia; as in 2009). It is a prospective, multicenter, standardized and anonymized database. Every trauma patient admitted to one of the participating trauma hospitals with an injury severity score (ISS) ≥ 16 or ICU treatment is documented in the registry. Data is continuously entered into a web-based data server that is hosted by the German Trauma Society and its Academy for Trauma Surgery (AUC). Irreversible data anonymity is guaranteed both for the individual patients and the participating hospitals. The registry comprises epidemiologic, physiologic, laboratory, diagnostic, operative, interventional and intensive care medical data as well as scoring and outcome data [12].

We analyzed patients from the period from 01/01/2002-12/31/2011 for reasons of data homogenicity.

### Statistical analysis

All statistical analyses were performed using SPSS version 21. Patients entered into the registry who suffered an accident between 2002 and 2011 were analyzed.

After identification of the enclosure variable “thoracic trauma”, the primary end-point was set as “in-house hospital mortality” to divide the collective in two dichotome collectives. Then, logistic regression models and stepwise forward selection were calculated to identify independent and dependent variables which influence the primary outcome variable. The influence of various factors on the target-variable duration of ventilation was analyzed by using multiple linear regression and stepwise forward selection as was described before [13] (inclusion p < 0.01).

For the demographic section, we have subdivided the study collective in different subgroups and have made a dichotome analysis of mortality of the collectives respective of their division (age, gender).

## Results

### Part I: Patient demographics

Within the observation period from 2002–2011, more than 93000 patients were recorded in the trauma registry of the TraumaRegister DGU® (TR-DGU). From these, 22613 patients fulfilled the primary study variable “thoracic trauma” and were enrolled into further statistical analysis.

The mean age was 46.1 years and 74.7% of the patients were male. Detailed information concerning demographics is depicted in Table 1.

**Table 1:** Demographics of study patients

	**Mean**	**Standard deviation**
**Age**	46.1	19.7
**ISS**	30.6	12.6
**Days of ventilation**	7.3	11.5
**Days admission to ICU**	11.7	14.1
**Days admission to hospital**	25.3	25.0
**Mean RR on admission**	116.7	34.8

The table shows the demographic properties of the overall study population that met the inclusion criteria.

### Mechanism of injury

The most frequent mechanism of injury in the overall study population was Road Traffic Accidents (RTAs). Overall, RTAs made up for 56.0% of mechanisms of injury with car crashes being the most frequent cause among RTAs (36.4%) followed by motorcycle crashes (16.8%) and injured pedestrians (7.4%). Falls made up for most of the remaining injuries (overall 27.4%; see Table 2).

**Table 2:** Mechanisms of injury in the study population

	**Percent of study population**
**Car passenger**	35.6
**Motorcyclist**	16.5
**Cyclist**	6.5
**Pedestrian**	7.3
**Fall >3 m**	19.6
**Fall <3 m**	7.3
**Others**	5.0
**Total**	**100**

The table shows the relative proportions of the mechanisms of injury in the study population as documented in the Traumaregister.

### Mortality after blunt thoracic trauma is influenced by age and gender

The outcome following Blunt Thoracic Trauma has been analyzed by forming 4 groups of patients. Patients in groups aged <55 years (mortality 13.5%) and between 55–64 years (15.7%) are less likely to die following blunt chest trauma than the average patient (17.5%). Patients aged 65–74 years (24.0% mortality) and over 75 years of age (40.0%) are severely at risk (see Table 3).

**Table 3:** Outcome following blunt chest trauma depending on age the table shows mortality dependent on age in different subgroups of the overall study population

**Age**	**Survived**	**Died**	**Total**
**< 55 years**	13055	2034	15089
	86.5%	13.5%	100%
**55-64 years**	2301	429	2730
	84.3%	15.7%	100%
**65-74 years**	1953	616	2569
	76.0%	24.0%	100%
**≥75 years**	1336	889	2225
	60.0%	40.0%	100%
**Total**	18645	3968	22613
	82.5%	17.5%	100%

Male patients not only sustained a severe thoracic injury more often. Also, they suffered a lower mortality (16.5%) following blunt chest trauma than female patients (20.5%; see Table 4).

**Table 4:** Outcome following blunt chest trauma depending on gender the table shows mortality dependent on gender in the two subgroups of the overall study population

	**Survived**	**Died**	**Total**
**Female**	4525	1166	5691
	79.5%	20.5%	100%
**Male**	13998	2773	16771
	83.5%	16.5%	100%
**Total**	18523	3969	22462
	82.5%	17.5%	100%

### Influence of extrathoracic injuries on mortality

The prognostic impact of extrathoracic injuries has been quantified in the most relevant AIS categories head, abdomen and extremity injuries Less severe injuries have no negative impact on mortality as compared to patients without injuries of the respective body regions in all groups. Injuries with AIS of ≤ 3 in all groups showed better rather than adverse mortality. AIS scores of 4 or greater in all extrathoracic regions however are correlated with mortality in a linear fashion (see Table 5).

**Table 5:** Influence of extrathoracic injuries on mortality following blunt chest trauma

	**Head**			**Abdomen**			**Extremity**		
**AIS**	**Survived**	**Died**	**Total**	**Survived**	**Died**	**Total**	**Survived**	**Died**	**Total**
**0**	7295	1034	8239	10558	2284	12842	4771	1306	6077
	87.6%	12.4%	100%	82.2%	17.8%	100%	78.5%	21.5%	100%
**1**	1331	64	1395	54	3	57	352	38	390
	95.4%	4.6%	100%	94.7%	5.3%	100%	90.3%	9.7%	100%
**2**	2509	196	4027	4123	497	4620	6359	949	7308
	92.8%	7.2%	100%	89.2%	10.8%	100%	87.0%	13.0%	100%
**3**	3521	506	4027	2208	427	2635	5164	872	6036
	87.4%	12.6%	100%	83.8%	16.2%	100%	85.6%	14.4%	100%
**4**	2504	519	3012	1168	396	1564	1707	535	2242
	82.8%	17.2%	100%	74.7%	25.3%	100%	76.1%	23.9%	100%
**5**	1441	1437	2878	532	355	887	292	268	560
	50.1%	49.9%	100%	60.0%	40.0%	100%	52.1%	47.9%	100%
**6**	44	212	256	2	6	8	n.a.	n.a.	n.a.
	17.2%	82.8%	100%	25.0%	75.0%	100%			

### Prevalence of thoracic injuries in the study group

The prevalence of common thoracic injuries was quantified in the study population and the results are depicted in Table 6.

**Table 6:** Frequency of injuries (% are rounded up if above 1%)

**Injury**	**n = (% of patients)**
Lung contusion	10864 (48%)
Pneumothorax	8878 (39%)
Rib fractures	7794 (35%)
Hemothorax	6223 (28%)
Flail chest	3681 (16%)
Lung laceration	2644 (12%)
Sternal fracture	1947 (8%)
Thoracic vessel injuries	633 (3%)
Cardiac injury	171 (0.8%)

The relative frequencies of the respective injuries have been investigated in the overall study population and the result is shown in Table 6.

Simple rib fractures of one or more ribs were a common injury and are found in roughly a third of patients, while flail chest is less common; sternal fractures are encountered only in about every tenth patient.

Lung contusion is common in patients with blunt thoracic injuries and found in about half of the patients; lung parenchymal injury on the other hand is comparatively rare and found only in one in ten patients. The occurrence of hemothorax was common as were pneumothoraces. Injuries to thoracic vessels or cardiac injuries are infrequent.

### Part II: Influence of thoracic injuries on survial

We next sought to identify which factors actually determine survival in severely injured patients after blunt thoracic trauma. Therefore, we performed univariate analysis with forward selection to identify which diagnoses actually pose a severe threat to survival in these severely injured patients (Table 7).

**Table 7:** Influence of extrathoracic factors and thoracic diagnoses on survival (factors with significantly adverse prognosis are written in bold letters)

	**Regression coefficient B**	**Significance p=**	**Exp(B) = odds ratio**	**95% CI for OR**	
				**Upper**	**Lower**
Thoracic vessel injuries		.000			
Vessel injuries AIS 2	.554	.310	1.740	.597	5.071
Vessel injuries AIS 3	.913	.000	2.492	1.522	4.081
Vessel injuries AIS 4	-.020	.927	.980	.634	1.513
**Vessel injuries AIS 5**	**1.683**	**.000**	**5.382**	**3.669**	**7.894**
**Vessel injuries AIS 6**	**2.864**	**.000**	**17.531**	**7.688**	**39.976**
Lung contusions		.000			
Unilateral minor (AIS 2)	-.577	.000	.562	.474	.665
Unilateral major (AIS 3)	-.264	.000	.768	.682	.865
**Bilateral (AIS 4)**	**.374**	**.000**	**1.454**	**1.226**	**1.723**
Lung lacerations		.000			
Unilateral minor (AIS 3)	-.280	.015	.756	.603	.948
Unilateral major or bilateral minor (AIS 4)	-,103	,290	,902	,745	1,092
**Bilateral major (AIS 5)**	**1,114**	**,000**	**3,047**	**2,061**	**4,505**
Rib fractures		,000			
One rib (AIS 1)	-,018	,895	,983	,758	1,273
Two ribs (AIS 2)	-,558	,000	,572	,467	,702
Three or more (AIS 3)	-,338	,000	,713	,630	,808
Flail chest		,000			
Unilateral minor (AIS 3)	-,089	,374	,914	,751	1,114
Unilateral minor (AIS 3)	-,091	,408	,913	,737	1,132
**Bilateral (AIS 5)**	**,530**	**,000**	**1,698**	**1,401**	**2,059**
Patient age		,000			
**Age 55-64**	**,424**	**,000**	**1,527**	**1,313**	**1,777**
**Age 65-74**	**,970**	**,000**	**2,638**	**2,298**	**3,028**
**Age 75+**	**1,940**	**,000**	**6,957**	**6,100**	**7,934**
Pneumothorax/Hemopneumothorax		,000			
Minor Pneumothorax/NFS (AIS 2)	-,332	,000	,718	,596	,864
Minor Hemopneumothorax (AIS 3)	-,309	,000	,734	,626	,861
Major Hemothorax or Pneumothorax (AIS 4)	-,050	,475	,952	,830	1,090
Tension (AIS 5)	,136	,187	1,145	,936	1,401
Blood transfusion		,000			
**1-9 Units**	**,598**	**,000**	**1,819**	**1,628**	**2,033**
**10 + Units**	**1,289**	**,000**	**3,630**	**3,108**	**4,239**
**Initial systolic pressure < 90**	**,695**	**,000**	**2,004**	**1,804**	**2,227**
**Systolic pressure < 90 upon admission**	**1,106**	**,000**	**3,023**	**2,706**	**3,377**
**AIS Head ≥ 3**	**1,367**	**,000**	**3,922**	**3,552**	**4,332**
**AIS Abdomen ≥ 3**	**,314**	**,000**	**1,368**	**1,222**	**1,533**
Sternal fracture	-,228	,008	,796	,672	,943
**Structural heart injury (AIS ≥ 3)**	**1,502**	**,000**	**4,491**	**2,894**	**6,970**
Constant	−3,519	,000	,030		

The variables influencing the target variable mortality following blunt thoracic trauma have been investigated using logistic regression analysis and forward selection for each variable as documented in the registry.

The only thoracic wall injury that was significantly associated with elevated mortality was bilateral flail chest, which had a moderately elevated Odds Ratio. None of the other chest wall injuries was significantly predictive of increased mortality.

Neither hemothoraces nor pneumothoraces themselves were associated with adverse prognoses regarding mortality, suggesting that even tension pneumothorax is usually recognized and treated successfully. Lung injuries such as contusions or lacerations were only predictive if major or bilateral. Injuries to thoracic vessels or heart injuries were severe injuries with drastically impaired chances of survival.

### Part III: Impact of extrathoracic factors additional to thoracic injuries on ventilator days

In order to analyze the influence of various factors on the duration of ventilation following blunt chest trauma, ventilation days were analyzed. Only patients who survived the first 14 days following injury were investigated using multivariate linear regression analysis (as demonstrated in Table 8). Our analysis identified a plethora of injuries as well as extrathoracic factors that prolong ventilation.

**Table 8:** Influence of extrathoracic factors and thoracic injuries on duration of ventilation in the study group as indicated in ventilator days

	**Non standardized coefficients**	**Standardized coefficients**	**Significance p=**
(constant)	−1,183		,000
Age 50–59 years	1,368	,040	,000
Age 60–69 years	2,584	,066	,000
Age 70–79 years	3,746	,086	,000
Age over 80 years	2,887	,047	,000
Male patient	1,327	,047	,000
AIS Head ≥ 3	5,821	,234	,000
AIS Abdomen ≥ 3	1,912	,064	,000
AIS Extremity ≥ 3	1,317	,052	,000
Shock (RR ≤90) upon resuscitation	2,027	,063	,000
Shock (RR ≤90) upon admission	3,052	,079	,000
Blood transfusion (under 10 units)	4,458	,153	,000
Blood transfusion (over 10 units)	5,447	,093	,000
Hemothorax minor (AIS 3)	1,094	,032	,000
Hemothorax major (AIS 4)	2,649	,069	,000
Flail chest unilateral minor (AIS 3)	,823	,016	,029
Flail chest unilateral major (AIS 4)	,813	,015	,036
Flail chest bilateral (AIS 5)	3,927	,062	,000
Unilateral minor lung laceration (AIS 3)	2,525	,046	,000
Unilateral major or bilateral minor lung laceration (AIS 4)	4,543	,086	,000
Bilateral major lung laceration (AIS 5)	5,658	,034	,000
Lung contusion unilateral minor (AIS 3)	1,331	,050	,000
Lung contusion major (AIS 4)	3,643	,072	,000
Cardiac laceration (AIS ≥ 4)	8,814	,045	,000
Pneumothorax severe without tension (AIS 4)	1,089	,031	,008
Tension pneumothorax (AIS 5)	3,033	,050	,000
Major intrathoracic vessel lesion (AIS 5/6)	6,231	,036	,000

Extrathoracic factors known to generally cause increased mortality also prolong ventilation in our study (age over 65 years, blood transfusion, shock, extrathoracic severe injury).

Some injuries to the thoracic wall that do not have a significant effect on mortality such as unilateral flail chest are independent predicting factors of prolonged ventilation following blunt chest trauma in our study collective, but only cause moderate prolongation. Nearly every type of injury to the lung parenchyma (lung contusions as well as lung laceration) statistically prolongs ventilation with a linear correlation of injury severity to the expected prolongation.

While the presence of a hemo- or pneumothorax in a patient following severe chest trauma is not predictive of mortality, hemo- and severe pneumothoraces are significant predictors of prolonged ventilation, albeit to a moderate degree.

Major thoracic vessel- and cardiac injuries not only pose highly lethal injuries in our analysis, but also drastically prolong ventilation in survivors.

## Discussion

Based on the analysis of a collective of 22613 patients with severe chest injury from the database of the TraumaRegister DGU®, we demonstrate for the first time the influence of specific structural damages of thoracic organs on the mortality of patients regarding their risk of mortality. Furthermore, we demonstrated the quantitative effect of specific injuries on the duration of ventilation in survivors using multiple linear regression and stepwise forward selection.

Blunt thoracic trauma is a common form of injury in severely injured patients and a frequent cause of mortality and morbidity in severely injured patients [1],[2],[5],[14].

A number of previous studies have addressed the prognosis of patients with blunt thoracic trauma. Pape et al. devised a scoring system (the Thoracic Trauma Score TTS) from observations in a series of 1495 trauma patients with blunt chest injury with the aim of having early guidance for clinical decision-making. They found that injuries to the parenchymal organs showed greater correlation to mortality and prolonged ventilation than chest wall injuries [4]. This finding is so far consistent with the data obtained in our study, which also indicates a significantly adverse prognosis after blunt thoracic trauma if the parenchymal organs are affected.

Many reports have addressed the consequences of single injuries on the prognosis following blunt chest trauma. The mortality of rib fractures following chest trauma has already been extensively studied with controversial findings. However, there is a consensus that rib fractures show increased mortality in the elderly [15],[16] and that a higher number of fractured ribs correlates with adverse outcome [17],[18].

Our study results show that older patients have drastically inferior outcome following blunt chest trauma with rib fractures and are well in line with previous findings. However, due to the nature of the AIS classification, which is used to enter and analyze diagnoses in the TraumaRegister DGU®, all rib fractures of three or more ribs are analyzed in the same group. However, we found no effect of rib fractures on survival or ventilation. Nevertheless, the overall effect of rib fractures on mortality remains controversial following our study, given that a recent meta-analysis identified three or more rib fractures as a predictor of mortality while another retrospective large study has not found rib fractures to be an independent prognostic factor in 35416 patients [14],[19].

Only few reports have addressed the prognosis of sternal fractures; without additional injuries they are described to have a rather benign prognosis [20],[21]. Consistent with these findings, we did not identify the diagnosis of a sternal fracture to be an independent predictor of morbidity or mortality.

Flail chest injuries have more increased mortlity than other chest wall injuries in our study. While only bilateral flail chest is associated with increased mortality, all forms of flail chest were found to be predictive of prolonged ventilation. Bilateral flail chest has previously been found to be a severely threatening condition compared to unilateral flail chest [22]. The rather benign prognosis of unilateral flail chest has been confirmed by a study of 262 cases where an isolated unilateral flail chest was associated with a mortality of about 6% [23].

Besides the mortality of chest wall injuries, the outcomes of the diverse intrathoracic injuries have been previously addressed in a number of studies. Pulmonary contusion seems to contribute to the likelihood and length of mechanical ventilation [8],[9]. Especially the notion that the likelihood and length of ventilation correlate with the extent of lung contusion matches the findings of our study. In our study, limited lung contusion does not have an effect but extensive lung contusion is associated with an increased risk of mortality and elevated mean duration of ventilation. However, we report on the outcome of a collective of over 10000 patients with different degrees of lung contusion following blunt chest trauma for the first time in the present study.

Previous reports on the impact and outcome of pulmonary laceration following blunt chest trauma are scarce. Two Japanese studies of 13 and 42 cases report mortality rates as high as 44% following pulmonary lacerations [24],[25]. However, we detected lung lacerations in 12% of patients with severe blunt chest trauma making the diagnosis of a lung laceration a very common finding. The high frequency of lung lacerations is most likely mediated by the frequent use of CT scans in recent years. In our study, only bilateral lung laceration was an actually independent risk factor for mortality following blunt chest trauma, while lung lacerations were common. On the other hand, all forms of blunt chest trauma are predictive of longer ventilation.

Previous reports on intrathoracic vessel injuries and cardiac lacerations have already suggested that these are associated with high mortality [7],[26]. Not surprisingly, we found these the most lethal injuries following blunt chest trauma and as suggested by long ventilation they cause significant morbidity in survivors.

The use of computed tomography (CT) scans in polytrauma patients is emerging in the last years with recent evidence hinting at a benefit in early survival after initial use of CT [13]. The use of CT for the evaluation of patients with blunt chest trauma has been common practice for 15 years and yields more diagnoses than common chest X-rays [10],[11]. However, the initially treating physician is confronted with an ever-increasing number of possible diagnoses following the first evaluation after blunt chest trauma using CT. Treatment decisions such as definite care versus damage control operations depend on the correct evaluation of the individual patient’s risk profile [27] and should be accurately based on evidence. The issue has been addressed before, but with less emphasis on the individual injuries encountered in these patients [2]. Based on the analysis of over 20000 patients with blunt chest trauma, our study provides physicians with evidence as to which injuries affect survival and cause prolonged ventilation. Future studies have to be conducted based on the current data to transform this into potential changes of action for clinical practice.

The limitations of the study arise mostly from its methodology. The study is strictly retrospective. Furthermore, diagnoses are only identified by their AIS codes, thus no absolutely strict description of each individual patient’s injuries is given. Furthermore, the study leaves out the duration of ventilation in patients who died in the first 14 days following trauma, thus creating a potential bias.

## Conclusions

We demonstrate quantitatively the influence of specific structural damages of the chest on critical outcome parameters. While most injuries of the chest wall have no or only limited impact in the study collective, injuries to the lung overall show adverse outcome. Injuries to the heart or thoracic vessels have a devastating prognosis following blunt chest trauma.

## Competing interests

The authors declare that they have no competing interest.

## Authors’ contribution

SH and RL analyzed the data set. SH, MvG and SHW planned the study and subgroups. RL performed statistical analysis. PD, HT, HW and PB contributed to study design and completion of the manuscript. All authors read and approved the final manuscript.
